# A Suggested New Bacteriophage Genus, “Kp34likevirus”, within the *Autographivirinae* Subfamily of *Podoviridae*

**DOI:** 10.3390/v7041804

**Published:** 2015-04-07

**Authors:** Harald Eriksson, Barbara Maciejewska, Agnieszka Latka, Grazyna Majkowska-Skrobek, Marios Hellstrand, Öjar Melefors, Jin-Town Wang, Andrew M. Kropinski, Zuzanna Drulis-Kawa, Anders S. Nilsson

**Affiliations:** 1 Department of Molecular Biosciences, the Wenner-Gren Institute, Stockholm University, Stockholm 106 91, Sweden; E-Mails: harald.eriksson@su.se (H.E.); marios.hellstrand@gmail.com (M.H.); 2 Institute of Genetics and Microbiology, University of Wroclaw, Wroclaw 51-148, Poland; E-Mails: barbara-boczkowska@wp.pl (B.M.); agnieszkalatka1989@gmail.com (A.L.); grazyna.majkowska-skrobek@uni.wroc.pl (G.M.-S.); zuzanna.drulis-kawa@uni.wroc.pl (Z.D.-K.); 3 Department of Microbiology, Public Health Agency of Sweden, Solna 171 82, Sweden; E-Mail: ojar.melefors@ki.se; 4 Department of Microbiology, National Taiwan University, College of Medicine, Taipei 10051, Taiwan; E-Mail: wangjt@ntu.edu.tw; 5 Departments of Food Science, Molecular & Cellular Biology, and Pathobiology, University of Guelph, Guelph, ON N1G 2W1, Canada; E-Mail: kropinsk@queensu.ca

**Keywords:** “Kp34likevirus”, *phikmvlikevirus*, *Autographivirinae*, *Klebsiella pneumoniae*, bacteriophage, phage, *Podoviridae*

## Abstract

*Klebsiella pneumoniae* phages vB_KpnP_SU503 (SU503) and vB_KpnP_SU552A (SU552A) are virulent viruses belonging to the *Autographivirinae* subfamily of *Podoviridae* that infect and kill multi-resistant *K. pneumoniae* isolates. Phages SU503 and SU552A show high pairwise nucleotide identity to *Klebsiella* phages KP34 (NC_013649), F19 (NC_023567) and NTUH-K2044-K1-1 (NC_025418). Bioinformatic analysis of these phage genomes show high conservation of gene arrangement and gene content, conserved catalytically active residues of their RNA polymerase, a common and specific lysis cassette, and form a joint cluster in phylogenetic analysis of their conserved genes. Also, we have performed biological characterization of the burst size, latent period, host specificity (together with KP34 and NTUH-K2044-K1-1), morphology, and structural genes as well as sensitivity testing to various conditions. Based on the analyses of these phages, the creation of a new phage genus is suggested within the *Autographivirinae*, called “Kp34likevirus” after their type phage, KP34. This genus should encompass the recently genome sequenced *Klebsiella* phages KP34, SU503, SU552A, F19 and NTUH-K2044-K1-1.

## 1. Introduction

In the post-genomic era there is a substantial number of phage genomes being deposited in NCBI GenBank, and the understanding of phages and their relationship to each other is growing. Early taxonomical classifications mainly focused on morphological similarities and the composition of the nucleic acid of the phage, be it double stranded or single stranded RNA or DNA. With the increased availability of fast and cheap next generation sequencing, the number of sequenced phages has increased drastically, which has also increased the need of having accurate taxonomical groups that are easily classified and understood. This process has begun and has resulted in the classification of the subfamilies of *Autographivirinae* and *Picovirinae* in the *Podoviridae* family, based on comparison of conserved genes [1]. New phage genera are established and generally named after the first fully sequenced phage within each classification, as with the genera within the *Autographivirinae* subfamily, the *T7likevirus* [2], the *Sp6likevirus* [3] and the *Phikmvlikevirus* [4]. These genera share the defining *Autographivirinae* ability to self-transcribe its genes, an ability acquired with the single-subunit RNA polymerase, common gene arrangement and common transcriptional scheme.

Phages KP34 [5] and NTUH-K2044-K1-1 [6] are *Podoviridae* within the subfamily of *Autographivirinae* and have been loosely associated with the *Phikmvlikevirus* genus. Both infect the Gram-negative, rod shaped, encapsulated and facultative anaerobic organism *Klebsiella pneumoniae*. This bacterium is an opportunistic pathogen which is often the cause of nosocomial infections and community acquired pneumonia [7]. In this study, two new phages have been isolated that both belong to the subfamily of *Autographivirinae*, and bear close resemblance to *Klebsiella* phages KP34 [5], NTUH-K2044-K1-1 [6] and F19 (NC_023567). These phages show significant nucleotide identity, a conserved recognition and specificity loop of the single-subunit RNA polymerase and a highly similar lysis cassette. In the traditional taxonomic classifications, organisms are grouped into natural groups based on easily observed characteristics, which has moved from the earlier observations purely based on morphological features, into more precise groups based on features of their genomes, transcriptional regime and gene content. Based on these findings, the creation of a new bacteriophage genus is suggested, called “Kp34likevirus”, named after the type phage KP34.

## 2. Materials and Methods

### 2.1. Biological Materials

Two phages, vB_KpnP_SU503 (SU503) and vB_KpnP_SU552A (SU552A), were isolated from the bioactive and aerobic step in the waste water treatment process of the Henriksdal waste water treatment plant situated in Stockholm, Sweden. Phages were isolated according to Pieroni *et al.* with some modifications [8]. One hundred mL of pre-centrifuged effluent was mixed with 100 mL double strength LB-broth and 10 mL of a 2 h, mid-logarithmic *K. pneumoniae* culture. Phage SU503 was isolated and propagated on *K. pneumoniae* 07RAFM-KPN-503 and SU552A on 07RAFM-KPN-552. Both are extended spectrum β-lactamse (ESBL) positive clinical isolates collected by The Public Health Agency of Sweden (Folkhälsomyndigheten) in 2007 [9]. Phage KP34 was propagated on *Klebsiella* isolate KPN77 and NTUH-K2044-K1-1 on NTUH-K2044 (Table S1).

Phage titer and plaque morphology was investigated using the standard soft agar overlay method [10] and performed simultaneously using the same conditions (medium, temperature and time of incubation). KP34 was isolated in Wroclaw, Poland [5], while *Klebsiella* phage NTUH-K2044-K1-1 was isolated in Taipei City, Taiwan (Table 1) [6].

**Table 1:** Bacteriophages used in this study.

Phage name	Reference	Country of origin	NCBI accession no.
vB_KpnP_SU503	this work	Sweden	KP708985
vB_KpnP_SU552A	this work	Sweden	KP708986
*Klebsiella* phage KP34	[5]	Poland	GQ413938
*Klebsiella* phage F19	[11]	China	KF765493
NTUH-K2044-K1-1	[6]	Taiwan	AB716666

### 2.2. Electron Microscopy

A filtered high-titer phage lysate was centrifuged at 25,000 *g* for 60 min. The pellet was washed twice in ammonium acetate (0.1 M, pH 7.0). Phages were deposited on copper grids with carbon-coated Formvar films (Sigma-Aldrich Co., St. Louis, MO, USA) and stained for 10 sec with uranyl acetate (2%, pH 4.5). Excess liquid was blotted off and phages were examined using a Zeiss EM 900 electron microscope (Carl Zeiss Microscopy GmbH, Jena, Germany) in Laboratory of Microscopy Techniques, University of Wroclaw, Poland. The magnification was calibrated using T4 phage tail length (114 nm) as a standard.

### 2.3. Burst Size Experiments

A one-step growth curve of isolated *Klebsiella* phages was performed according to the method of Pajunen *et al.* [12] with some modifications previously described [13].

### 2.4. Sensitivity of Phage Particles to Temperature, Chloroform and pH

Sensitivity of phage particles to temperature, chloroform and pH was evaluated as previously described [13]. Viruses were suspended in phosphate-buffered saline (PBS) (NaCl 137 mmol/L, KCl 2.7 mmol/L, Na_2_HPO_4_ 10 mmol/L, KH_2_PO_4_ 1.8 mmol/L, the pH adjusted to 7.4 with HCl and NaOH). Phage particles in PBS pH 7.4 were used as control. An equal volume of filter-sterilized bacteriophage of 10^8^ plaque-forming units per mL (PFU/mL) was mixed with chloroform and incubated for 2 h at room temperature (RT) with intermittent shaking. Further preparations of phages were incubated at pH 2, 4, 5, 6, 8 and 10 for 1 h and 5 h, at RT and 37 °C. A phage preparation was also incubated at 60 °C for 10 min. Phage titers were then assessed using the soft agar overlay method [10].

### 2.5. Determination of Phage Bacterial Host Range

The host range of the bacteriophages was determined on the 26 clinical *K. pneumoniae* strains listed in Table S2 [14]. Bacteria were stored at −70 °C in Trypticase Soy Broth (TSB) (Becton Dickinson and Company, Cockeysville, MD, USA) supplemented with 20% glycerol (Avantor Performance Materials Poland S.A., Gliwice, Poland). Spot testing was used as a rapid and efficient method for determining the host range in large collections of bacteria [14]. The phage titer used for spot testing was 10^5^ PFU/mL with final 5 × 10^2^ PFU per spot. A majority of the clinical isolates used in the host range analysis were selected from ESBL-positive *K. pneumoniae* strains isolated in Sweden during the year of 2007, individually picked for the largest variation of PFGE patterns (using XbaI) by the Public Health Agency of Sweden (Folkhälsomyndigheten) (Solna, Sweden).

### 2.6. Phage Structural Protein Analysis

Phage particles were purified by cesium chloride density gradient centrifugation method [15]. Phage structural protein analysis was performed by SDS-PAGE gel electrophoresis using Mini-Protean^®^TGX Stain-Free Precast Gels (Bio-Rad Laboratories, Inc., Hercules, CA, USA) [15]. Analysis of protein bands was performed using Molecular Imager^®^ Gel Doc™ XR System (Bio-Rad Laboratories, Inc., Hercules, CA, USA) and Quantity One^®^ software (Bio-Rad Laboratories, Inc., Hercules, CA, USA). Precision Plus Protein™ Unstained Standard (Bio-Rad Laboratories, Inc., Hercules, CA, USA) was used as a size marker for the molecular analysis of the phage structural proteins.

### 2.7. DNA Purification

Phage stocks (~10^9^ PFU/mL) were sterile filtered (0.45 µm Whatman Puradiscs, GE Healthcare Bio-Sciences, Pittsburgh, PA, USA) and DNase I and RNase A treated (Thermo Fischer Scientific, Waltham, MA, USA) for one hour at 37 °C to remove bacterial DNA and RNA contamination prior to phage genome recovery. Subsequent Proteinase K treatment (Thermo Fischer Scientific, Waltham, MA, USA) released phage DNA for purification using phase lock gel Heavy (5 Prime Inc., Gaithersburg, MD, USA) with phenol:chloroform:isoamyl alcohol (Sigma-Aldrich Co., St. Louis, MO, USA) as organic phase in accordance to manufacturer protocols. DNA was then ethanol precipitated using standard protocols [10].

### 2.8. DNA Sequencing

Phages SU503 and SU552A were sequenced using Roche 454 FLX+ titanium pyrosequencing technology (454 Life Sciences, Branford, CT, USA). The sequencing run resulted in 10506 reads from SU503 and 57583 reads from SU552A. Ends were trimmed prior to *de novo* assembly. Roche Newbler (2.6, 454 Life Sciences, Branford, CT, USA, 2011) was used to *de novo* assemble the reads produced by the sequencing run, and contigs smaller than 1000 nucleotides were discarded. From the SU503 sequencing run, 92.09% of the reads assembled into one singular large contig of 43,809 nucleotides with an average coverage of 86 reads per nucleotide. From SU552A, 97.7% of the reads assembled into a singular large contig of 43,594 nucleotides with an average coverage of 509 reads per nucleotide. Genome ends were identified by sequence homology to phage KP34 (GQ413938) and separated.

### 2.9. In Silico Analysis and Annotation of Genomes

The genomes of SU503 and SU552A were annotated using the RAST engine in Glimmer 3.0 mode [16] and with MetaGeneAnnotator [17] in parallel. Overlapping open reading frames (ORF) were detected by manually investigating each ORF for upstream Shine-Dalgarno sequence, start codon usage and ORF overlap length [18].

All open reading frames were compared to the nucleotide collection database (nr/nt) using BLASTN and translated amino acid sequences were searched for homologous proteins using PSI-BLAST in the non-redundant protein sequences database (nr) [19,20]. To investigate possible transfer-RNAs in the phage genomes, tRNAscan-SE was employed [21]. Putative Rho-independent terminators were identified using ARNold (http://rna.igmors.u-psud.fr/toolbox/arnold/index.php) with minimal energy threshold value set to −15.0 [22,23].

Novel phage promoters were discovered by extracting 100 nucleotides before each putative ORF using ExtractUpstreamDNA (https://lfz.corefacility.ca/extractUpStreamDNA/) and running it through the Multiple RM for Motif Elicitation (MEME) [24]. The same procedure was then performed by only extracting 100 nucleotides before each putative operon. Motifs were then manually investigated in each phage genome and conserved nucleotides marked.

The same annotation procedure was performed on phages KP34, F19 and NTUH-K2044-K1-1 (Table 1) to establish a common annotation framework. Ambiguous and conflicting ORF lengths were harmonized for overlaps, length and ribosomal binding sites.

### 2.10. Comparative Genomics

To establish a set of conserved genes between the genera within the subfamily of *Autographivirinae*, CoreGenes 3.5 was utilized on the sequenced genomes of type phages from each phage genus [25]. These phages were φKMV (NC_005045), SP6 (NC_004831), T7 (NC_001604) and KP34 (NC_013649). Determination of conserved genes between the *Phikmvlikevirus* and “Kp34likevirus” members were made through comparison of φKMV, LKA1 (NC_009936), KP34 and NTUH-K2044-K1-1. Linear genome visualization and tblastx [19] comparison was performed using Easyfig 2.1 [26] with a minimum length of BLAST hits set to be drawn to 30.

A local BLAST database was setup in Geneious (6.1.8, Biomatters Ltd., Auckland, New Zealand, 2013) containing the amino acid sequences inferred from the nucleotide sequences of the predicted open reading frames of phages SU503, SU552A, KP34, NTUH-K2044, F19 for pairwise identity comparison [27]. The datasets used in the phylogenetic analyses were assembled by comparison of the amino acid sequences of the genes, common to these five phage genomes, to the annotated proteins of other phages from the *Autographivirinae* subfamily. The amino acid sequences of three proteins corresponding to phage KP34 RNA polymerase (KP-KP34p37), head-tail connector protein (KP-KP34p40) and DNA maturase B (KP-KP34p50) were selected for phylogenetic analysis in MEGA6 [28]. In all, 33 phages were shown to harbor homologs to these genes, and the corresponding annotated protein sequences (28) were downloaded from GenBank. The alignments of the amino acid sequences were performed with MUSCLE [29]. The datasets contained 693 positions in the analysis of the RNA polymerase, and 342 in both the head-tail connector protein and DNA maturase B protein sequences datasets.

Phylogenetic analyses were performed in MEGA6 [28] using the maximum likelihood (ML) method based on the Whelan and Goldman substitution model [30]. Gaps or missing data were completely deleted and uniform substitution rates among sites selected in the analyses. Initial starting trees for the ML searches were autogenerated by the maximum parsimony (MP) method. The ML searches were carried out with the Nearest-Neighbor-Interchange (NNI) heuristic method, and the robustness of the resulting trees was evaluated by 500 bootstrap replicates.

### 2.11. Analysis of Lysis Cassette

Identification and *in silico* analysis of the lysis proteins and lysis cassette has been done using protein and nucleotide BLAST analysis [19], biosequence HMMER analysis (using profile hidden Markov models) [31], TOPCONS (identifying the presence of transmembrane domains) [32] and ExPASy SIB Bioinformatics Resource Portal [33].

## 3. Results

### 3.1. Phage Isolation and Morphological Characteristics

Phages SU503 and SU552A were isolated from the aerated bioactive stage in the Henriksdal waste water treatment plant. This waste water treatment plant receives effluent from both domestic and commercial sources but also from a major hospital in the Stockholm region, the Stockholm South General Hospital (Södersjukhuset). SU503 was isolated on clinical *K. pneumoniae* 07RAFM-KPN503 and SU552A on 07RAFM-KPN-552 (Table S1).

**Figure 1:**
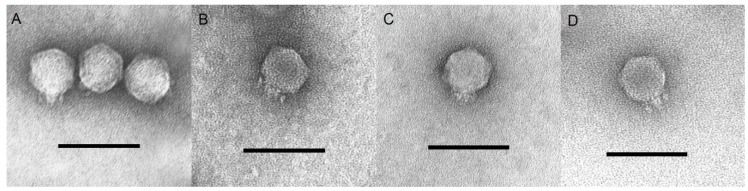
Transmission electron micrographs of (**A**) SU503; (**B**) SU552A; (**C**) KP34; (**D**) NTUH-K2044-K1-1. Length bar is 100 nm.

The bacteriophages SU503 and SU552A were examined simultaneously with NTUH-K2044-K1-1 and KP34 by transmission electron microscopy (TEM) and classified on the basis of their morphological features to be members of the *Podoviridae* family (Figure 1). The icosahedral heads of these four tested phages were the same size and estimated to be approximately 63 nm between opposite apices and the head was connected to a short 15 nm tail. Twenty six *K. pneumoniae* strains (Table S1) were used to determine the specificity of the four tested bacteriophages: SU503, SU552A, NTUH-K2044-K1-1 and KP34. The host range of NTUH-K2044-K1-1 was limited to the host strain. Both phages SU503 and SU552A were found to infect two out of 26 clinical isolates (Table S2). By comparison, phage KP34 showed lytic activity against eight of these strains (31%).

Using the soft agar overlay method, it was shown that phages SU552A and NTUH-K2044-K1-1 form small, clear plaques (about 1–2 mm diameter), whereas the remaining two phages (SU503 and KP34) produced turbid plaques with a diameter of 5 mm, on the strain used for their isolation. All plaques were surrounded by a hazy halo zone, indicating the ability of these phages to produce virion-associated polysaccharide-degrading enzymes [34]. The presence of a gene encoding pectate lyase has been confirmed in NTUH-K2044-K1-1 genome (Orf34) [6]. Homologous depolymerase genes have been identified in the other phages assessed in this study using *in silico* analysis, at locus tags KP-KP34p57, SU503_53, SU552A_54 and F19_51. The proteins encoded by mentioned genes are relatively large in size (55-70 kDa) and have dual functions, as a structural component of the tail fiber and enzymatic as polysaccharide-degrading enzyme. Their enzymatic activity according to software algorithms are probable pectate lyase. According to the one-step growth curves, all phages have a comparable latent period (15–20 min), but differ in respective average burst size. Phages KP34 and NTUH-K2044-K1-1 have approximately 50 virions per cell while SU503 and SU552A has less than half of that, approximately 20 virions per cell. These phage life cycle characteristics are in accord with the values observed for phage KP34 [5].

### 3.2. Physicochemical Properties

All phages were shown to be relatively sensitive to high temperature, with a 2-log (KP34 particles) or 3-log (remaining phages) decrease in titer observed after 10 min at 60 °C. While phage KP34 retained almost 100% infectivity after chloroform treatment, the activity reduction of other phages were by two orders (NTUH-K2044-K1-1) or three orders (SU503 and SU552A) of magnitude. Similar variation in chloroform sensitivity has been observed for *Klebsiella* phages KP15 and KP27, where closely related *Myoviridae* representatives showed different stability in chloroform treatment [13]. Such phenomenon could be explained by the observation that despite the general absence of lipids, about one third of tailed phages are chloroform-sensitive [35]. The optimal pH was determined by testing the stability of phages at different pH after 1 h and 5 h of incubation at RT and 37 °C. All tested phages lost their infective ability completely at pH 2.0 after 5 h incubation at both temperatures. One-hour incubation at the same acidity caused three and seven log decrease in phages SU503 and NTUH-K2044-K1-1, respectively. Phages SU552A and KP34 were found to be more sensitive to acidic conditions and completely lost its activity after 60 min at pH 2.0. Exposure to pH 4 at 37° C caused ten-fold PFU reduction of SU552A, SU503 and NTUH-K2044-K1-1, after 5 h incubation. In contrast, KP34 was less resistant to this pH value, where three and seven log decrease of titer was noticed after 1 and 5 h, respectively. All tested phages were stable within a pH range of 5–10 at both temperatures.

### 3.3. Analysis of Structural Proteins

**Figure 2:**
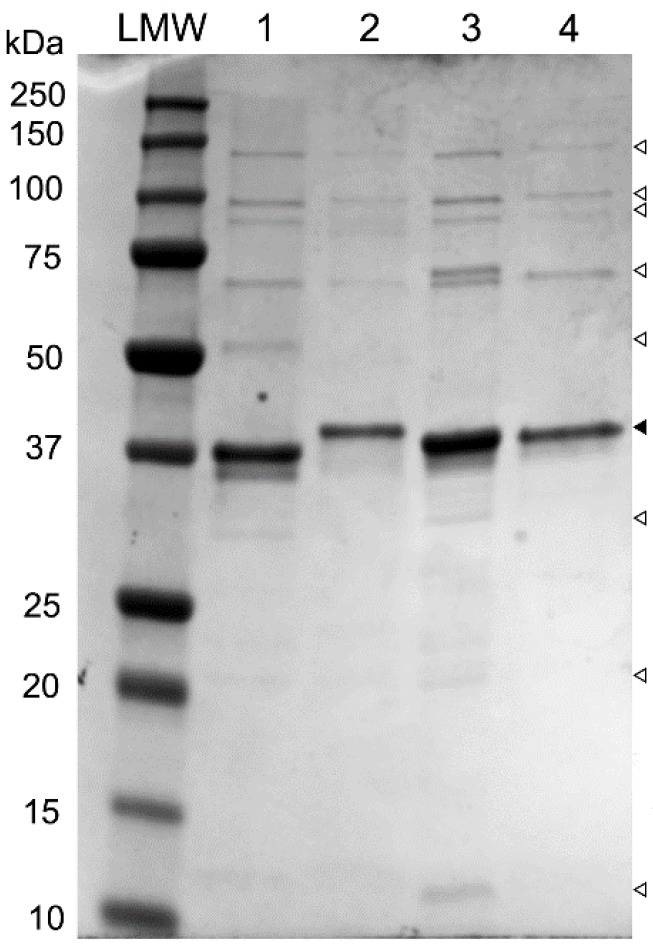
SDS-PAGE analysis of phage structural proteins. Lane LMW, reference ladder (kDa; BioRad), lane 1, phage SU503; lane 2, phage SU552A; lane 3, phage NTUH-K2044-K1-1; lane 4, phage KP34. ▼: solid arrow indicate major protein band; ∇: blank arrows show minor protein bands.

Comparative analysis of the genome sequences of phages SU503 and SU552A reveals the presence of seven genes that encode putative structural proteins which share high amino acid sequence identity to the other “Kp34likeviruses”: KP34, F19 and NTUH-K2044-K1-1, ranging from 86%–99%. By contrast, pairwise BLAST comparison of these same structural proteins with their φKMV equivalent, only shows an average of 30% (between 24%–38%) in pairwise identity. To further characterize these structural genes, an SDS-PAGE was performed. A major protein band and several minor protein bands were observed on the gel, with molecular weights ranging from approximately 20 to 135 kDa (Figure 2).

The predominant protein band at 37 kDa on the SDS gel are suggestive of major capsid protein, as it was shown earlier for phage KP34 [5]. The major capsid protein has at least 93% of sequence homology in phages KP34, F19 and NTUH-K2044-K1-1, which are all members of the proposed “Kp34likevirus” genus. The amino acid similarities of these capsid proteins, corresponding to the 37.7 kDa bands, compared to the homologous protein of phage φKMV are all less than 37%. In addition, other observed proteins bands could be correlated with KP34 structural proteins: the head-tail connector protein, the tail tubular proteins A and B, the internal virion protein B, the internal core protein, and the tail fiber protein.

### 3.4. Genome Analysis

Comparison of the phages within the suggested “Kp34likevirus” genus show high pairwise nucleotide identity, similar G+C content and genome size. Coding sequences (CDSs) in the described phages vary from 52 CDS in phage F19 to 57 CDS in phage KP34 (Table 2). SU503 has a genome size of 43,809 nucleotides and a G+C content of 53.7%. SU503 has a total of 55 predicted CDSs where 50 start with ATG start codons, two with TTG and three with GTG. SU552A has 56 predicted CDSs, with 51 ATG, three TTG and two GTG start codons. All phages within the proposed “Kp34likevirus” genus show large pairwise identity to each other (Table 2).

**Table 2:** Genome information and pairwise identity of the members of “Kp34likevirus”.

Phage	Size (bp)	G+C content, %	CDS	Nucleotide pairwise identity, %		
				KP34	SU503	SU552A	NTUH-K2044-K1-1	F19
KP34	43,809	54.1	57	-	78.6	79.3	77.5	77.5
SU503	43,809	53.7	55	78.6	-	75.1	78.1	76.8
SU552A	43,594	54.2	56	79.3	75.1	-	76.4	76.6
NTUH-K2044-K1-1	43,871	54.2	54	77.5	78.1	76.4	-	76.1
F19	43,766	53.8	52	77.5	76.8	76.6	76.1	-

Host transcription of the phage genomes starts at predicted sigma 70 promoter sequences, located upstream from the first predicted operon (Table 3). A putative phage-specific promoter was discovered on two locations in the phage genomes, corresponding to the early-middle genes in the phage genomes. This conserved motif shows high conservation across all “Kp34likevirus” member phages (Table 3), and is placed in intra genic regions of all phages. Since no promoter sequence have been identified before the late genes, this conserved motif cannot be solely responsible for phage specific transcription initiation. An alternative function of this sequence motif could be the location of single-stranded nicks that have been found in *Phikmvlikevirus* relative phage φkF77 [36]. An Rho-independent transcription terminator is located downstream of the conserved motif, located in between the genes corresponding to the structural genes of KP34, genes KP-KP34p43 and KP-KP34p44. It shares this terminator with the other phages of “Kp34likevirus” (Table 3). Sequences encoding tRNAs were not found in the genomes.

**Table 3:** Regulatory sequences of the phages in the suggested “Kp34likevirus” genus. Conserved motifs are presented in bold while underlined nucleotides depict loops.

Host Promoter		
Phage	location	nucleotide sequence
KP34	931..959	**TTGACA**CCGCGAAGAA**CATAAG**
SU503	1040..1068	**TTGACA**CCGCGAAGGACATAAGC**TAGATT**
	1169..1196	**TTAAAA**TAAACGCTTGACAAGT**TATGAT**
	1182..1210	**TTGACA**AGTTATGATTCACTGAG**TAACTT**
SU552A	683..710	**TTGCCC**TGCTTACCATTTTTGC**TATAAG**
	874..902	**TTGACA**CCGCGAAGAACATAAGC**TAGATT**
	943..971	**TTGACA**CCGCGAAGAACATAAGC**TAGATT**
NTUH-K2044-K1-1	953..981	**TTGACA**CCGCGAAGGACATAAGC**TAGATT**
	1022..1050	**TTGACA**AGTTCTGATTCACTGAG**TAACTT**
	1251..1280	**CTCACA**GGTTAGCAGTCCTGAGCC**GATAAG**
F19	968..996	**TTGACA**CCGCGAAGGACATAAGC**TAGATT**
	1051..1079	**TTGACA**AGTTCCGATTCACTGAG**TAACTT**
	1192..1220	**ACGACA**AACGGCGGGTGCGCTTA**GATGAT**
*E. coli* consensus sequence		TTGACA-(N15-18)-TATAAT
**Putative phage promoters**		
**Phage**	**location**	**nucleotide sequence**
KP34	1487..1538	CACTAATTAC**AGCCTATAGCATCCTACGGGGTGCTATGTGAA**GTAATTACCT
	2508..2559	TTTAGTAGCA**AGCCTATAGCGTCCTATGGGGCGCTATGTGAA**TGCAACTGGC
SU503	2010..2061	CGTTAATTAC**AGCCTATAGCATCCTACGGGGTGCTATGTGAA**GTAATTACCT
	3192..3243	TCCAGTAGCA**AGCCTATAGCGTCCTACGGGGCGCTATGTGAA**TGCAACTGGC
SU552A	1449..1500	CGTTAATTAC**AGCCTATAGCATCCTACGGGGTGCTATGTGAA**GTAATTACCT
	2225..2276	TATAGTAGCA**AGCCTATAGCGTCCTACGGGGCGCTATGTGAA**TGCAACTAGC
NTUH-K2044-K1-1	1752..1803	TACTAATTAC**AGCCTATAGCATCCTATGGGGTGCTATGTGAA**GTAATTACCT
	2618..2669	TCTAGTAGCA**AGCCTATAGCGTCCTACGGGGCGCTATGTGAA**TGCAACCGGC
F19	1795..1846	CGTTAATTAC**AGCCTATAGCATCCTATGGGGTGCTATGTGAA**GTAATTACAT
	2571..2622	TATAGTAGCA**AGCCTATAGCGTCCGACTGGGCGCTATGTGAA**TGCAACTAGC
Phage promoter consensus		**AGCCTATAGCGTCCTACGGGGCGCTATGTGAA**
**Rho-independent terminators**		
**Phage**	**location**	**nucleotide sequence**
KP34	26623..26660	GCCCCTGGTGCCTTCGGGTGCCAGGGGCTTTTTTTTTT
SU503	26936..26972	GCCCCTGGTGCCTTCGGGTGCCAGGGGCTTTTTTTTT
SU552A	25384..25420	GCCCCTGGTGCCTTCGGGTGCCAGGGGCTTTTTTTTT
NTUH-K2044-K1-1	25925..25961	GCCCCTGGTGCCTTCTGGTGCCGGGGGCTTTTTTTTT
F19	26363..26400	GCCCCTGGTGCCTTCGGGTGCCAGGGGCTTTTTTTTTT

### 3.5. Comparative Genomics

**Figure 3:**
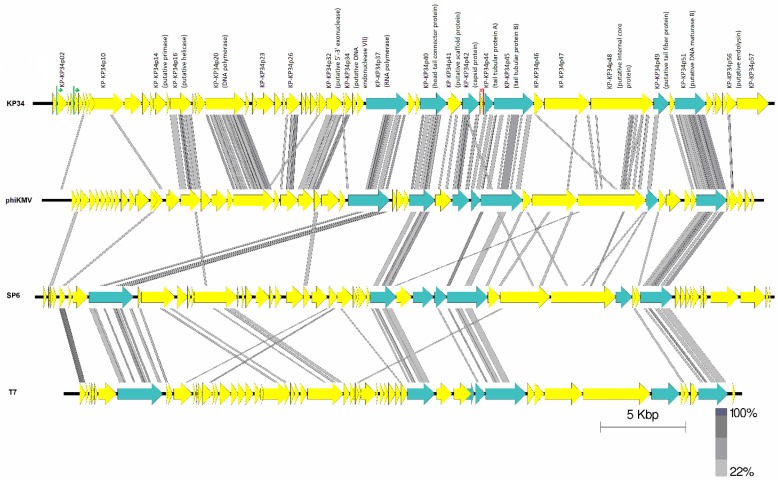
Pairwise comparison using tblastx of the four type phages KP34, φKMV, SP6 and T7. Lines between genomes represent high amino acid similarity between the phages using tblastx analysis. Blue arrows denotes conserved genes across the *Autographivirinae* subfamily. Green lines show putative phage specific promoter sites and red rho-independent terminator in phage KP34.

A total of seven conserved proteins were identified, using CoreGenes 3.5 (Figure 3) [25], among phages within the subfamily of *Autographivirinae*. These included the DNA-dependent RNA polymerase, DNA maturase A and B, head-tail connector protein, the tail tubular protein A and B and the major capsid protein gene.

Between phages of the genera *Phikmvlikevirus* and phages from the suggested “Kp34likevirus” there are 18 conserved proteins. A total of 29 genes are conserved between the phages within the “Kp34likevirus” genus (Table 4).

**Figure 4:**
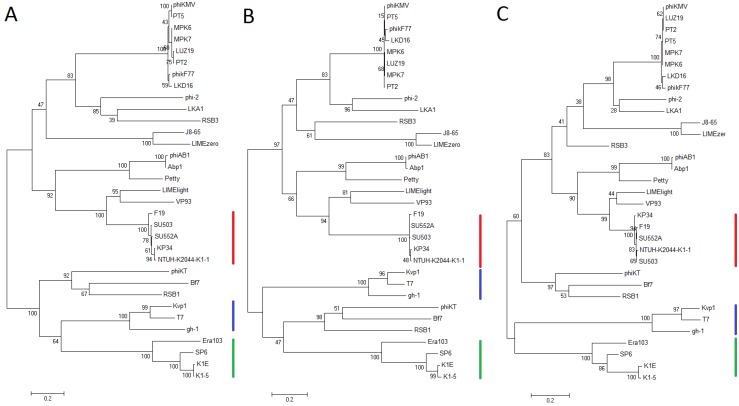
The phylogenetic analyses of amino acid sequences, inferred from three genes from 33 phages in the *Autographivirinae* subfamily, were performed by using the maximum likelihood (ML) method based on the Whelan and Goldman model [30], with 500 bootstrap replicates. The bootstrap percentages are shown next to each node. Initial trees for the searches were autogenerated by the maximum parsimony (MP) method. The following ML searches were performed using uniform substitution rates among sites and the Nearest-Neighbor-Interchange (NNI) heuristic search method. Positions containing gaps and missing data were excluded. The trees with the highest likelihoods are drawn to scale, with branch lengths measured in the number of substitutions per site. Red lines indicate the “Kp34likevirus” cluster, blue *T7likevirus* members and green *SP6likevirus* members. *Phikmvlikevirus* members’ φKMV, LKA1, LIMElight and LIMEzero are scattered in the upper clade of the phylograms. The analyses were conducted in MEGA6 [28]. (**a**) The RNA polymerase tree (log likelihood = −26580.7487); (**b**) The head-tail connector protein tree (log likelihood = −12447.9348); (**c**) The DNA maturase B (terminase) tree (log likelihood = −10492.0745).

To investigate the evolutionary relationship between phages from the “Kp34likevirus”, *Phikmvlikevirus*, and the more distant *Sp6likevirus* and *T7likeviruses* within the *Autographivirinae* subfamily, phylogenetic analyses were performed on the amino acid residues of the RNA polymerase, head-tail connector protein and the DNA maturase B protein (Figure 4). In all three phylograms the phages of the proposed “K34likevirus” cluster into a single monophyletic clade. This division is also conserved when analyzing the other conserved proteins from phages of the *Autographivirinae* subfamily, the major capsid protein, tail tubular protein A and B and DNA maturase A. The whole genome nucleotide identity against the two phages in the sister group, consisting of the *Vibrio* phage VP93 and the *Pantoea* phage LIMElight, was shown to be only 9% and 15% respectively.

**Table 4:** Conserved genes among phages within the “Kp34likevirus” and their amino acid pairwise identity to phage KP34. Conserved genes between KP34 and φKMV shown in bold. ^a^: a.a. query coverage range from 84.7%–85.04%. ^b^: a.a. query coverage range from 91.31%–99.06%. ^c^: a.a. query coverage range from 50.0%–76.43%.

No.	Description	KP34 Locus_Tag	Accession no.	% Pairwise AA identity to KP34			
				SU503	SU552A	K2044	F19
1	hypothetical protein	KP-KP34p02	GI:282554636	96.8	97.9	94.1	97.9
2	hypothetical protein	KP-KP34p05	GI:294661414	63.0	65.8	64.4	65.8
3	hypothetical protein	KP-KP34p10	GI:282554643	84.0 ^a^	89.0 ^a^	88.3 ^a^	87.9 ^a^
4	putative peptidase	KP-KP34p11	GI:282554645	87.4	97.1	98.0	98.0
5	hypothetical protein	KP-KP34p12	GI:282554646	68.8	70.5	62.7	67.1
**6**	**putative DNA primase**	**KP-KP34p14**	**GI:282554648**	**93.6**	**95.8**	**95.0**	**96.5**
**7**	**putative DNA helicase**	**KP-KP34p16**	**GI:294661415**	**96.0 ^b^**	**98.6**	**97.7**	**97.7 ^b^**
8	**DNA polymerase**	**KP-KP34p20**	**GI:282554654**	**81.5**	**84.1**	**81.8**	**80.8**
9	hypothetical protein	KP-KP34p23	GI:282554657	88.1	81.9	85.8	72.9
**10**	**hypothetical protein**	**KP-KP34p26**	**GI:282554660**	**96.5**	**98.7**	**96.5**	**95.8**
11	hypothetical protein	KP-KP34p29	GI:282554662	78.5	83.5	91.8	86.0
**12**	**putative 5'-3' exonuclease**	**KP-KP34p32**	**GI:282554665**	**93.5**	**92.2**	**91.9**	**92.5**
**13**	**putative DNA endo- nuclease VII**	**KP-KP34p34**	**GI:294661421**	**99.3**	**94.3 ^c^**	**51.4 ^c^**	**98.6**
**14**	**DNA-dependent RNA polymerase**	**KP-KP34p37**	**GI:282554612**	**96.8**	**96.4**	**98.4**	**94.6**
15	hypothetical protein	KP-KP34p38	GI:282554613	97.9	90.4	89.7	91.1
**16**	**head-tail connector protein**	**KP-KP34p40**	**GI:294661422**	**98.9**	**98.9**	**99.2**	**98.3**
**17**	**putative scaffolding protein**	**KP-KP34p41**	**GI:282554617**	**98.2**	**98.2**	**97.9**	**97.5**
**18**	**capsid protein**	**KP-KP34p42**	**GI:282554619**	**92.9**	**93**	**93.5**	**92.7**
**19**	**tail tubular protein A**	**KP-KP34p44**	**GI:282554621**	**87.9**	**91.9**	**97.7**	**92.2**
**20**	**tail tubular protein B**	**KP-KP34p45**	**GI:282554622**	**99.0**	**98.0**	**96.2**	**88.4**
21	putative internal virion protein B	KP-KP34p46	GI:282554623	75.4	98.5	75.9	99.0
**22**	**hypothetical protein**	**KP-KP34p47**	**GI:282554624**	**96.1**	**95.0**	**92.6**	**88.0**
**23**	**putative internal core protein**	**KP-KP34p48**	**GI:282554625**	**97.4**	**97.9**	**89.0**	**70.2**
**24**	**putative tail fiber protein**	**KP-KP34p49**	**GI:282554626**	**93.8**	**65.1**	**90.3**	**92.5**
**25**	**putative DNA maturase A**	**KP-KP34p50**	**GI:282554627**	**97.0**	**100**	**100**	**100**
**26**	**putative DNA maturase B**	**KP-KP34p51**	**GI:282554628**	**98.7**	**98.9**	**98.1**	**98.9**
27	hypothetical protein	KP-KP34p52	GI:282554629	97.4	98.4	98.4	97.6
28	hypothetical protein (spanin)	KP-KP34p54	GI:282554631	98.5	97.8	97.0	96.3
**29**	**putative endolysin**	**KP-KP34p56**	**GI:282554633**	**90.6**	**96.0**	**93.3**	**89.8**

### 3.6. RNA Polymerase

Three structurally important areas in the DNA-dependent RNA polymerase protein sequence were analyzed, the catalytically essential residues Asp537, Lys631, Tyr639 and Asp812, the recognition loop between amino acids 93 to 101 and the specificity loop between amino acids 739 to 770 [4,37,38]. All catalytically essential amino acid residues are conserved within all RNA polymerases, both in φKMV and KP34-like viruses, but there is a large divergence of the recognition loop, where the residues are identical amongst the KP34-likeviruses (Table 5). The specificity loop shows conservation within the “Kp34likevirus”, with substitutions in residues 751 to 755. Phages NTUH-K2044-K1-1 and KP34 have an identical specificity loop and phages F19, SU503 and SU552A are identical to each other.

**Table 5:** Alignment of the recognition and specificity loops of the RNA polymerase in φKMV, “Kp34likevirus”. Phages VP93 (NC_012662) and LIMElight (NC_019454), which assembled close to the “Kp34likevirus” members in the phylogenetic analysis are added for comparison. Underlined peptides show sites of substitutions compared to KP34.

Phage	Recognition loop	Specificity loop
φKMV	HQEAKAAKPAAKL	EEVRVRLRAEAVEYVTLYEAK-DEL
KP34	MRNVKAPGIGGKY	EEVRVRIDCMNLSAVLVHNRDFKTC
K2044	MRNVKAPGIGGKY	EEVRVRIDCMNLSAVLVHNRDFKTC
F19	MRNVKAPGIGGKY	EEVRVRIDCMNLTIMRVHNRDFKTC
SU503	MRNVKAPGIGGKY	EEVRVRIDCMNLTIMRVHNRDFKTC
SU552A	MRNVKAPGIGGKY	EEVRVRIDCMNLTIMRVHNRDFKTC
LIMElight	IKAEKAPGVGGKY	EEKRVNIRSMGLTQVVAYNRNYDLN
VP93	LKASKTRGVGAKY	HETRVKVRSMGINQVVLYNFDYERN

### 3.7. Lysis Cassette

Through genomic analysis of all five “Kp34likevirus” members: KP34, SU503, SU552A, NTUH-K2044-K1-1 and F19, a total of three proteins involved in the host lysis have been identified. These enzymes act jointly in the sequential order: holin, endolysin and spanin. The holins are small, hydrophobic proteins, able to form pores in the inner membrane allowing endolysins (peptidoglycan hydrolases) to gain access to the periplasm and attack the bonds in the murein structure [39,40,41,42]. Instead of a classic holin, bacteriophages may encode a variant, the pinholin, capable of forming small pores with a diameter too narrow to transfer endolysins but wide enough for ion movement and membrane depolarization, thereby for signal arrest release (SAR) or signal peptide (SP) endolysin activation [40,43]. The spanins acts at the end of the lysis process leading to the destruction of the outer membrane and phage progeny release.

The mentioned lysis proteins have been identified and characterized through *in silico* analysis of the “Kp34likevirus” genomes. At the outset, it is worth noting that the amino acid sequences of these enzymes are highly similar, over 95%. *In silico* analysis of each genome revealed holin genes with characteristic features; (i) a small size of about 83 residues; (ii) positively charged hydrophilic domain at the C-terminus; (iii) one transmembrane domain of class III holins, (iv) a holin gene adjacent to the endolysin gene. The analysis of the endolysins genes in the investigated phages indicates that these enzymes possess a SAR domain at the N-terminus. The SAR signal identification follows the presence of two consecutive sections; positively charged initial section, containing lysine or arginine (or both), and a hydrophobic H-region [40]. Both elements have been identified for the analyzed endolysins encoded by the KP34-like viruses. The catalytic domain of these endolysins with lysozyme activity is located directly behind the H-region. The presence of a SAR signal within the endolysin gene indicates that the described holins are in fact pinholins responsible for the endolysin activation. Analysed spanins belong to the u-spanin group and are represented by a single protein with two transmembrane domains arranged at distal parts of the enzyme. Spanins may exist as an i-spanin/o-spanin complex of two proteins such as Rz and Rz1, or as a single acting unimolecular protein called u-spanin [44,45].

**Figure 5:**
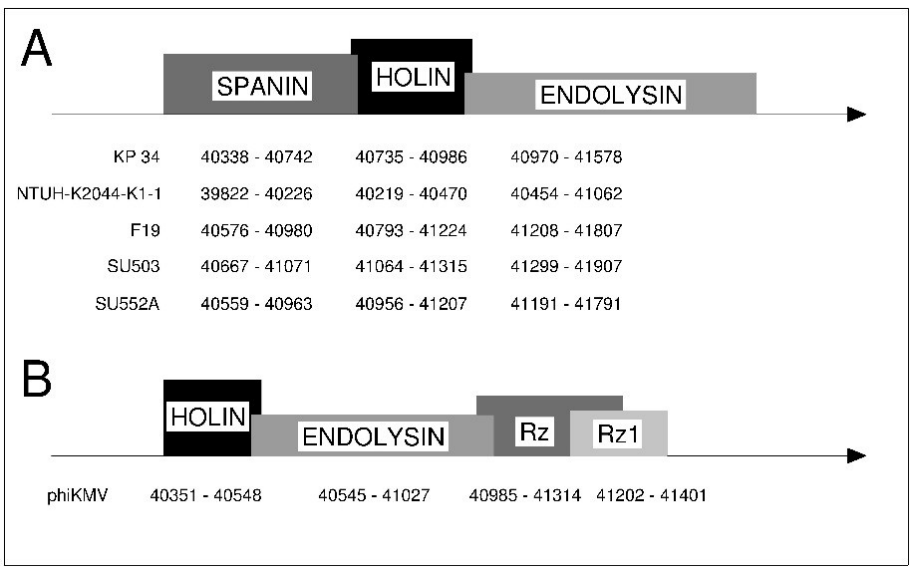
Lysis cassette scheme in the analyzed (**A**) “Kp34likevirus” and (**B**) φKMV.

The genes involved in lysis of KP34-like viruses are located next to each other (successively: spanin –holin–endolysin) and thereby form a “lysis cassette” (Figure 5A). The presence and organization of a lysis cassette that encodes a holin, endolysin, Rz and Rz1, in this particular order, has been previously described for phage φKMV (Figure 5B) [40]. However, there are major differences between the lysis cassette of φKMV and “Kp34likevirus”. These differences relate to the amino acid sequences, length, gene organization, and spanin construction. In contrast to φKMV, the lysis cassette of KP34-like viruses is formed from a unimolecular spanin, holin and endolysin, respectively.

Compared to KP34, the amino acid sequence similarity of the lysis cassette is 96% (at a coverage 100%) for NTUH-K2044-K1-1, 93% (with 98% coverage) for F19, 93% (with 98% coverage) for SU503 and 97% (with 99% coverage) for SU552A, whereas the similarity of the φKMV to the KP34 lysis cassette is about 30% (22% coverage). The low similarity of lysis cassettes indicates a distant relationship between these genes in phage φKMV and the “Kp34likevirus” member phages.

## 4. Discussion

In this article we propose the creation of the new phage genus, “Kp34likevirus”, based on the high pairwise identity of the investigated phage genomes, the use of a single tail fiber gene, the highly conserved RNA polymerase recognition sites and specificity loops, as well as conserved lysis cassette genes. The “Kp34likevirus” genus is suggested to encompass *Klebsiella* phages KP34, SU503, SU552A, F19 and NTUH-K2044-K1-1.

At nucleotide level, the pairwise identity of phages KP34, SU503, SU552A, NTUH-K2044-K1-1 and F19 range from between 75.1 to 79.3% (Table 2). The similarity between the “Kp34likevirus” phages is even higher when comparing the pairwise identity of the amino acids of the gene products, with few exceptions (Table 4) and highly conserved order of genes between each other in comparison to φKMV. All phages within the “Kp34likevirus” genus have different host ranges, shown experimentally, which suggest that they utilize different host receptors (Table S2).

In contrast to the phages in the *Phikmvlikevirus* genus, the RNA polymerase responsible for transcribing the phage genes from phage specific promoters, show identical recognition loops between all the five phages, and a small variation of the specificity loop. The differences of the residues in the specificity loop divides them into two separate subgroups, with phage KP34 and NTUH-K2044-K1-1 in one group and phages SU503, SU552A and F19 in another (Table 5). The high identity of the specificity and recognition loops in their RNA polymerases suggest that the “Kp34likevirus” phages utilize a common promoter sequence, of which one has been predicted showing large conservation among the phages (Table 2).

Phylogenetic analysis of conserved genes places the “Kp34likevirus” phages in a monophyletic clade (Figure 4). In comparison to phages in the *Sp6likevirus* and *T7likevirus* genera, the division of phages into the *Phikmvlikevirus* and “Kp34likevirus” genera is a more recent occurrence in evolutionary terms from the *Autographivirinae* ancestor. Interestingly, the *Phikmvlikevirus* phage LIMElight and the unclassified phage VP93 closely affiliate to the suggested “Kp34likevirus” genus by phylogenetic analysis of the conserved genes, but while LIMElight shares the KP34 feature of a single tail fiber gene, it does not show any homology to the KP34 tail fiber genes, but shows instead larger homology to SP6 tail fiber genes. This has previously been suggested to be due to lateral acquisition of the tail fiber gene in this phage [46]. LIMElight has a slightly “Kp34likevirus”-like conserved recognition loop, and lesser conserved specificity loop of its RNA polymerase, and also a lysis cassette similar to the “Kp34likevirus” phages. The low nucleotide identity of LIMElight to KP34, 15%, also argues a distant relationship between the two phages.

*Vibrio* phage VP93, on the other hand, shows a clear divergence from the phages in “Kp34likevirus”, with dual tail fiber genes, no identifiable holin or endolysin, and no similarity in the RNA polymerase specificity and recognition loops (Table 5). The only commonality with the “Kp34likevirus” is the overall peptide similarity of the conserved genes across *Autographivirinae* (Figure 2). DNA pairwise identity of VP93 to KP34 is 9%. In depth analysis of the VP93 proteome using HHpred indicates that VPP93_gp41 encodes a protein structurally similar (E-value = 5.6 × 10^−25^) to a muramoyl-pentapeptide carboxypeptidase from *Streptomyces albus* (RCSB Protein Data Bank accession number: 1lbu). This protein shows weak similarity to gp46 (NP_853606) of *Enterobacteria* phage SP6.

The organization of the “Kp34likevirus” lysis cassette is based on spanin, holin and endolysin genes located sequentially in contrast to the phage φKMV lysis system (holin-endolysin-spanins sequence) (Figure 5). The differences were also detected in spanin structure where an i-spanin/o-spanin complex of two proteins is present in φKMV phage and u-spanin (unimolecular) exists in “Kp34likevirus” phages.

Taken together, the high nucleotide pairwise identity and amino acid similarity, promoter specificity and lysis cassette organization argues for the creation of the new *Autographivirinae* genus named “Kp34likevirus”, composed of phages KP34, SU503, SU552A, NTUH-K2044-K1-1 and F19. The phages belonging to this suggested genus have been collected from two different continents, and display a high degree of conservation. This genus will be proposed to the International Committee on Taxonomy of Viruses (ICTV) for ratification.

## Supplementary Files

Supplementary File 1
