# Supplementary material for: A Suggested New Bacteriophage Genus, “Kp34likevirus”, within the Autographivirinae Subfamily of Podoviridae
Source: Viruses. 2015 Apr 7;7(4):1804–22. doi: 10.3390/v7041804 (PMC4411677; doi:10.3390/v7041804)
Supplement: Supplementary File 1 [file viruses-07-01804-s001.pdf]

Supplemental Information

Table S1. *Klebsiella pneumoniae* strains used in this study.

Strain	Resistance mechanism	Antibiogram	PFGE type	Country of isolation	Refs.
07RAFM-KPN-501	ESBL-A CTX-M group 1	Gen	KP-05	Sweden	[9]
07RAFM-KPN-502	n (SHV wt)	Tmp	nt	Sweden	[9]
07RAFM-KPN-503	n (SHV wt)	Tmp	nt	Sweden	[9]
07RAFM-KPN-505	ESBL-A SHV S238	Tmp	S	Sweden	[9]
07RAFM-KPN-506	ESBL-A CTX-M group 9	Tmp	S	Sweden	[9]
07RAFM-KPN-507	ESBL-A CTX-M group 1, ESBL-A SHV S238	CipGenTmp	KP-03	Sweden	[9]
07RAFM-KPN-510	ESBL-A CTX-M group 9, ESBL-A SHV S238	CipGenTmp	KP-07	Sweden	[9]
07RAFM-KPN-511	ESBL-A CTX-M group 1	CipTmp	S	Sweden	[9]
07RAFM-KPN-512	ESBL-A CTX-M group 1	CipGenTmp	S	Sweden	[9]
07RAFM-KPN-513	ESBL-A CTX-M group 1, ESBL-A SHV S238+K240	CipTmp(Tob)	S	Sweden	[9]
07RAFM-KPN-514	ESBL-A CTX-M group 1	CipTmp(Tob)	KP-01	Sweden	[9]
07RAFM-KPN-515	n (SHV wt)	Tmp	nt	Sweden	[9]
07RAFM-KPN-524	ESBL-A CTX-M group 9	-	S	Sweden	[9]
07RAFM-KPN-525	n (SHV wt)	Tmp	nt	Sweden	[9]
07RAFM-KPN-527	ESBL-A CTX-M group 1	CipTmp	KP-01	Sweden	[9]
07RAFM-KPN-534	n (SHV wt)	-	S	Sweden	[9]
07RAFM-KPN-537	ESBL-A SHV S238+K240	CipTmp(Tob)	S	Sweden	[9]
07RAFM-KPN-542	ESBL-A CTX-M group 1	Tmp	S	Sweden	[9]
07RAFM-KPN-549	ESBL-M DHA	CipTmp(Tob)	S	Sweden	[9]
07RAFM-KPN-550	ESBL-A CTX-M group 1	Tmp	S	Sweden	[9]
07RAFM-KPN-552	ESBL-A SHV S238+K240	GenTmp	KP-02	Sweden	[9]
07RAFM-KPN-557	ESBL-A CTX-M group 1	-	KP-04	Sweden	[9]
07RAFM-KPN-559	ESBL-A CTX-M group 1	Tmp	S	Sweden	[9]
07RAFM-KPN-566	ESBL-A CTX-M group 1, ESBL-A SHV S238	Tmp	S	Sweden	[9]
KPN 77	-	-	-	Poland	[5]
NTUH-K2044-K1-1	-	-	-	Taiwan	[6]

All 07RAFM strains were isolated during 2007.

Table S2. Investigated host ranges of the "Kp34likeviruses".

Strain	SU503	SU552A	NTUH-K2044-K1-1	KP34
07RAFM-KPN-501	-	-	-	COL
07RAFM-KPN-502	-	-	-	COL
07RAFM-KPN-503	CCL	-	-	COL
07RAFM-KPN-505	-	-	-	COL
07RAFM-KPN-506	-	-	-	-
07RAFM-KPN-507	-	-	-	-
07RAFM-KPN-510	CCL	-	-	COL
07RAFM-KPN-511	-	-	-	COL
07RAFM-KPN-512	-	-	-	-
07RAFM-KPN-513	-	-	-	COL
07RAFM-KPN-514	-	-	-	-
07RAFM-KPN-515	-	COL	-	-
07RAFM-KPN-524	-	-	-	-
07RAFM-KPN-525	-	-	-	-
07RAFM-KPN-527	-	-	-	-
07RAFM-KPN-534	-	-	-	-
07RAFM-KPN-537	-	-	-	-
07RAFM-KPN-542	-	-	-	-
07RAFM-KPN-549	-	-	-	-
07RAFM-KPN-550	-	-	-	-
07RAFM-KPN-552	-	CCL	-	-
07RAFM-KPN-557	-	-	-	-
07RAFM-KPN-559	-	-	-	-
07RAFM-KPN-566	-	-	-	-
KPN 77	-	-	-	CCL
NTUH-K2044	-	-	CCL	-

CCL: cofluent clear lysis; COL: cofluent opaque lysis; dash: no lysis.
